# Region-Specific, Life-Threatening Diseases among International Travelers from Israel, 2004–2015

**DOI:** 10.3201/eid2404.171542

**Published:** 2018-04

**Authors:** Chen Avni, Shmuel Stienlauf, Eyal Meltzer, Yechezkel Sidi, Eli Schwartz, Eyal Leshem

**Affiliations:** The Center for Travel Medicine and Tropical Diseases, The Chaim Sheba Medical Center, Tel Hashomer, Israel; Sackler School of Medicine, Tel Aviv University, Tel Aviv, Israel

**Keywords:** posttravel hospitalization, vaccination, malaria, chemoprophylaxis, vaccination, life-threatening, prophylaxis, leishmaniasis, schistosomiasis, Salmonella enterica serovar Paratyphi, Staphylococcus, malaria, Plasmodium falciparum, Plasmodium vivax, dengue, leptospirosis, Salmonella enterica serovar Typhi, hepatitis, enteric infections, dengue, Caribbean, South America, Central America, Africa, Asia, Israel, India, Thailand, Ethiopia, vector-borne infections

## Abstract

We characterized posttravel hospitalizations of citizens returning to Israel by summarizing the returning traveler hospitalization dataset of the national referral Center for Travel Medicine and Tropical Diseases at Sheba Medical Center in Israel. Of 722 hospitalizations, 181 (25%) infections were life-threatening; most would have been preventable by chemoprophylaxis and pretravel vaccination.

International travel, particularly to tropical regions and low-income countries, may be associated with the risk for acute illness and hospitalization (*1*). The Center for Travel Medicine and Tropical Diseases at Sheba Medical Center (SMC; Tel Hashomer, Israel) is the national referral center for travel-associated illness in Israel. We characterized posttravel hospitalizations of citizens returning to Israel by summarizing the SMC returning traveler hospitalization dataset. 

## The Study

We investigated all international travel–associated hospitalizations of citizens of Israel at SMC during 2004–2015. We excluded case-patients for whom the time interval between return from travel and symptom onset exceeded the known incubation period for the cause of hospitalization. When identified illness after travel leading to hospitalization was nonendemic to Israel and caused symptoms after a long incubation (e.g., leishmaniasis, schistosomiasis), patients were included regardless of the time interval since return. We defined nonspecified febrile illness as a febrile illness with an undetermined cause (*2*). We excluded hospitalized persons who had unspecified febrile illness when the interval between return from travel and disease onset exceeded 2 weeks, because of the lower certainty of association between travel and illness. We defined acute and potentially life-threatening tropical diseases as infectious diseases largely confined to tropical and subtropical areas of the world that had an incubation period of >4 weeks and an estimated risk for death >5% within 4 weeks after symptom onset if left untreated (*3*).

We determined the country of disease acquisition by a history of travel to a single country or exposure to a single country during the incubation period for the cause of hospitalization. To put the number of hospitalizations for illness acquired in each destination country in context of the estimated number of Israelis traveling to that country, we extracted the number of Israeli citizen entries by country from the United Nations World Tourism Organization dataset (*4*). We compared continuous variables by using the Student *t*-test and compared categorical variables by using the χ^2^ test. Statistical significance was set at p<0.05. The SMC Institutional Review Board approved this study.

During 2004–2015, a total of 722 travelers returning to Israel were hospitalized (Table 1; Technical Appendix Table 1). The median patient age was 33 years (interquartile range 26–50 years); 530 (73%) were male. By continent, 330 (46%) patients had traveled to Asia; 267 (37%) to Africa; and 73 (10%) to South America, Central America, and the Caribbean. The travel destination countries from which the highest number of travelers were hospitalized were India (116 [16%]), Thailand (106 [15%]), and Ethiopia (48 [7%]). In relative terms, several countries, mostly in Africa, had a high number of hospitalizations respective to the estimated number of entries by Israeli citizens (Figure; Technical Appendix Table 2).

**Table 1:** Characteristics of travel-associated hospitalizations of citizens of Israel at Sheba Medical Center, Israel, 2004–2015*Category

Category	Africa, n = 267	Asia, n = 330	South America, n = 43	Central America/ Caribbean, n = 30	North America/ Europe, n = 26	Other,† n = 26	Total, n = 722
Patient characteristics							
Sex							
M	226 (85)	219 (66)	28 (65)	20 (67)	21 (81)	16 (62)	530 (73)
F	41 (15)	111 (34)	15 (35)	10 (33)	5 (19)	10 (38)	192 (27)
Age, median (IQR)	41 (29–53)	29 (24–43)	24 (23–43)	29 (27–46)	55 (39–64)	27 (23–41)	33 (26–50)
Age ≥60 y	37 (14)	27 (8)	4 (9)	3 (10)	8 (31)	4 (15)	83 (11)
Category of travelers							
Tourism	154 (58)	313 (95)	41 (95)	29 (97)	23 (88)	26 (100)	586 (81)
Business travelers	93 (35)	17 (5)	2 (5)	1 (3)	3 (12)	0	116 (16)
Visiting friends or relatives	19 (7)	1 (<1)	0	0	0	0	20 (3)
Type of illness							
Potentially preventable	85 (32)	25 (8)	0	1 (3)	0	1 (4)	112 (16)
Febrile conditions							
Malaria							
* Plasmodium falciparum*‡	82 (30)	4 (1)	0	0	0	0	86 (12)
* P. vivax*	23 (9)	7 (2)	3 (7)	0	0	3 (12)	36 (5)
* P. ovale*	8 (3)	0	0	0	0	0	8 (1)
* P. malariae*	7 (3)	0	0	0	0	0	7 (<1)
Unidentified malaria	6 (2)	2 (<1)	0	0	0	0	8 (1)
Dengue fever	4 (1)	59 (18)	2 (5)	8 (27)	0	1 (4)	74 (10)
Enteric fever							
* Salmonella enterica* serovar Typhi	2 (<1)	19 (6)	0	1 (3)	0	1 (4)	23 (3)
* S. enterica* ser. Paratyphi	0	36 (11)	0	0	0	0	36 (5)
Leptospirosis	1 (<1)	18 (5)	0	7 (23)	1 (4)	2 (8)	29 (4)
Pneumonia	10 (4)	11 (3)	0	1 (3)	5 (19)	1 (4)	28 (4)
Febrile diarrheal diseases	10 (4)	5 (2)	1 (2)	0	2 (8)	1 (4)	19 (3)
Acute schistosomiasis	16 (6)	3 (<1)	0	0	0	0	19 (3)
Influenza	0	7 (2)	0	0	0	1 (4)	8 (1)
Epstein–Barr virus	1 (<1)	6 (2)	1 (2)	0	0	0	8 (1)
Cytomegalovirus	4 (1)	2 (<1)	2 (5)	0	0	0	8 (1)
Amebic liver abscess	0	5 (2)	0	0	0	2 (8)	7 (<1)
Rickettsial diseases	3 (1)	3 (<1)	0	0	0	0	6 (<1)
Upper respiratory tract infection	4 (1)	1 (<1)	0	0	0	0	5 (<1)
Unspecified febrile illness	34 (13)	55 (17)	7 (16)	3 (10)	6 (23)	4 (15)	109 (15)
Other febrile conditions	15 (6)	29 (9)	8 (19)	2 (7)	3 (12)	2 (8)	59 (8)
Afebrile conditions							
Afebrile diarrheal diseases	5 (2)	7 (2)	2 (5)	0	4 (15)	0	18 (2)
Afebrile eosinophilia	4 (1)	6 (2)	2 (5)	1 (3)	1 (4)	2 (8)	16 (2)
Skin disease	7 (3)	4 (1)	2 (5)	1 (3)	0	0	14 (2)
Afebrile nondiarrheal GI illness	3 (1)	8 (2)	2 (5)	0	0	0	13 (2)
Viral hepatitis	2 (<1)	8 (2)	0	2 (7)	0	0	12 (2)
Leishmaniasis	2 (<1)	0	9 (21)	0	0	0	11 (2)
Giardiasis	0	4 (1)	0	1 (3)	0	0	5 (<1)
Other afebrile	14 (5)	21 (6)	2 (5)	3 (10)	4 (15)	6 (23)	50 (7)
Outcome							
Intensive care unit hospitalization	4 (1)	4 (1)	0	0	2 (8)	1 (4)	11 (2)
Death	1 (<1)	1 (<1)	0	0	0	0	2 (<1)

*Values are no. (%) patients except as indicated. Further details are available in online Technical Appendix Table 1 (https://wwwnc.cdc.gov/EID/article/24/4/17-1542-Techapp1.pdf). GI, gastrointestinal; IQR, interquartile range. †Other comprises case-patients in Oceania (n = 6) and those for whom exact region of infection was undetermined (n = 26). ‡One patient returning from Asia with malaria had a coinfection with *P. falciparum* and *P. vivax*. In this table, we listed the patient under *P. falciparum*.

**Figure:**
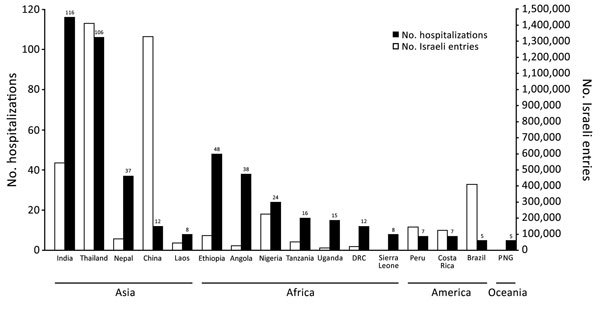
Travel-associated hospitalizations of citizens of Israel at Sheba Medical Center, Israel, by country of disease acquisition (A), and estimated number of Israeli citizen entries to each country (B), 2004–2005. Data on Israeli citizen entries from the United Nations World Tourism Organization (*4*). DRC, Democratic Republic of the Congo; PNG, Papua New Guinea.

Overall, the most common causes of hospitalization were malaria (145 [20%]), dengue (74 [10%]) and enteric fever (59 [8%]). Among 145 hospitalized malaria patients, 86 (59%) tested positive for *Plasmodium falciparum*. For Asia, the most common causes of admission were dengue fever, enteric fever, and unspecified febrile illnesses; for Africa, the most common were malaria, unspecified febrile illnesses, and acute schistosomiasis; and for South America, Central America, and the Caribbean, the most common were dengue fever and leptospirosis.

Patients hospitalized for *P. falciparum* malaria (n = 86) were older than those positive for *P. vivax* (n = 36) (43 ± 14 y vs. 34 ± 12 y; p<0.01) and were more likely to be business travelers (39 [45%] vs. 1 [3%]); p<0.01); male (81 [94%] vs. 26 [72%]; p<0.01); and to have traveled to middle and western Africa (64 [74%] vs. 0; p<0.01). The annual number of *P. vivax* malaria hospitalizations declined during the study period from an average of 7.3 hospitalizations per year during 2004–2006 to <1 hospitalization per year during 2013–2015 (R^2^ = 0.62).

Of the 181 acute life-threatening tropical diseases, 86 (48%) were acquired in Africa and 83 (46%) in Asia (Table 2). Male sex, business travel, and travel to Africa characterized travelers hospitalized for treatment of life-threatening diseases. The most common causes of life-threatening illness requiring hospitalization were *P. falciparum* malaria (86 [48%]) and enteric fever (59 [33%]). Of the 74 cases of dengue fever, none were dengue hemorrhagic fever or dengue shock syndrome; therefore, no dengue cases were considered life-threatening. Eleven (2%) hospitalized travelers required admission to an intensive care unit, and 2 of these patients died during their hospitalization. One patient died from endocarditis caused by *Staphylococcus aureus* and 1 from necrotizing fasciitis in a surgical wound while hospitalized for eosinophilia and abdominal mass.

**Table 2:** Comparison of characteristics of travelers hospitalized for treatment of acute life-threatening tropical diseases and those with non–life-threatening illnesses, Sheba Medical Center, Israel, 2004–2015*

Patient characteristics	Life-threatening illness,† n = 181	Non–life-threatening illness, n = 541	p value
Male sex	145 (81)	385 (71)	0.02
Age, median (IQR)	33 (25–49)	33 (25–50)	0.62
Elderly, age ≥60 y	15 (8)	68 (13)	0.12
Category of travelers			
Tourism	138 (76)	448 (83)	0.05
Business travelers	43 (24)	73 (13)	<0.01
Visiting friends or relatives	0 (0)	20 (3)	<0.01
Continent of travel			
Africa	86 (48)	181 (33)	<0.01
Asia	83 (46)	247 (46)	0.98
South America	0	43 (8)	<0.01
Central America and Caribbean	8 (4)	22 (4)	0.83
North America and Europe	1 (<1)	25 (5)	<0.01
Other‡	3 (2)	23 (4)	0.11

*Values are no. (%) patients except as indicated. IQR, interquartile range. †Life-threatening diseases (n = 181): *P. falciparum* (86), *S. enterica* ser. Paratyphi (36), leptospirosis (29), *S. enterica* ser. Typhi (23), rickettsial diseases (3), melioidosis (2), hantavirus (1), trypanosomiasis (1). ‡Other comprises Oceania (n = 6) and cases whose exact region of infection was undetermined (n = 26).

A total of 112 (16%) hospitalizations were potentially preventable by chemoprophylaxis or pretravel vaccination: *P. falciparum* malaria (86, 12%); *Salmonella enterica* serovar Typhi (23, 3%); hepatitis A (2, <1%); and acute hepatitis B (1, <1%). Most of the life-threatening diseases acquired in Africa were potentially preventable (85, 99%); significantly fewer (25, 30%) from Asia were potentially preventable (p<0.01).

## Conclusions

We reviewed >700 posttravel hospitalizations of citizens in Israel during 2004–2015. Acute, life-threatening illnesses necessitated 25% of admissions, most of which were potentially preventable by malaria chemoprophylaxis or pretravel vaccination. Compared with other regions, nearly all life-threatening diseases in travelers returning from Africa were preventable.

Most travelers who were admitted to hospitals to treat preventable life-threatening diseases after returning from Africa were diagnosed with *P. falciparum* malaria. The number of *P. vivax* malaria hospitalizations declined during the study years, possibly related to discontinuation of rafting trips to the Omo River in Ethiopia, which had resulted a high number of infections in earlier years. In travelers returning from Asia, enteric fever was the second most common cause of hospitalization, after dengue fever; *S. enterica* ser. Paratyphi, for which an effective vaccine is not available, caused most of those illnesses. An outbreak of *Salmonella* Paratyphi A enteric fever in Nepal (*5*) may have contributed to this trend.

Our study has several limitations. SMC is the national referral center for travel-related illness; therefore, an unusually high number of severe or complicated illnesses may have affected our results. Because of the relatively small number of hospitalizations related to individual destination countries, singular events or large outbreaks may have biased the country-specific data (*5*). The Israeli traveler population is generally characterized by a low rate of travelers visiting friends and relatives, except travelers to Ethiopia. Approximately one third of the patients hospitalized after travel to Ethiopia were born in Ethiopia or born to parents from Ethiopia who immigrated to Israel. This relationship may have resulted in a higher posttravel hospitalization number among citizens of Israel returning from Ethiopia, because travelers visiting friends and relatives may be at a higher risk (*6*). Because of the different methods of traveler data capture used by different countries reporting to the United Nations World Tourism Organization, our use of the reported number of Israeli citizen entries from this dataset was limited to contextualize the number of hospitalizations from specific countries in relative terms, rather than to calculate country-specific rates of hospitalization.

In conclusion, Israeli citizens hospitalized to treat life-threatening diseases after returning from travel to Africa were likely to suffer from preventable illnesses. Knowledge of region-specific hospitalization causes and impact should be used to identify at-risk travelers, enhance pretravel preparation, and advocate adherence to recommended vaccines and malaria prophylaxis.

Technical Appendix Detailed information on travel-associated hospitalizations by region of travel (Technical Appendix Table 1) and description of causes of hospitalization of Israeli citizens traveling to and returning from various countries (Technical Appendix Table 2).
