# Supplementary material for: Region-Specific, Life-Threatening Diseases among International Travelers from Israel, 2004–2015
Source: Emerg Infect Dis. 2018 Apr;24(4):790–3. doi: 10.3201/eid2404.171542 (PMC5875270; doi:10.3201/eid2404.171542)
Supplement: Technical Appendix — Detailed information on travel-associated hospitalizations by region of travel (Technical Appendix Table 1) and description of causes of hospitalization of Israeli citizens traveling to and returning from various countries (Technical Appendix Table 2). [file 17-1542-Techapp-s1.pdf]

Article DOI: https://doi.org/10.3201/eid2404.171542

Preventable Region-Specific Life-Threatening Diseases among Israeli International Travelers, 2004–2015

Technical Appendix

Technical Appendix Table 1 contains detailed information on all travel-associated hospitalizations included in the present study by region of travel.

In the description of causes of hospitalization acquired in a single destination country in Technical Appendix Table 2, we included travelers for whom the country of disease acquisition was determinable (634/722, 88%). We excluded countries in which <5 hospitalizations were recorded during the study years. Only countries with consistent entries reporting and ≥5 hospitalizations are shown (Technical Appendix Table 2).

We extracted the total number of Israeli citizen entries for each destination country during 2004–2015 from the United Nations World Tourism Organization dataset (*1*).In some countries, reporting of Israeli entries was inconsistent (i.e., there were years with missing entry data). Countries with consistent entry reporting were defined as countries which reported the number of Israeli citizen entries during all years or at least 11 out of 12 years included in the study.We included all countries with ≥5 hospitalizations regardless of whether they consistently reported to the United Nations World Tourism Organization; and when reporting was inconsistent, we denote as non-applicable (NA) for number of entries.

Technical Appendix Table 1. Travel-associated hospitalizations at Sheba Medical Center, Israel 2004–2015 (N = 722)*†

Category	Africa<br>n = 267 (%)	Asia<br>n = 330 (%)	South America<br>n = 43 (%)	Central America and the Caribbean<br>n = 30 (%)	North America and Europe<br>n = 26 (%)	Other§<br>n = 26 (%)	Total<br>N = 722 (%)
Patient characteristics							
Sex, male	226 (85)	219 (66)	28 (65)	20 (67)	21 (81)	16 (62)	530 (73)
Age, median (IQR)	41 (29–53)	29 (24–43)	24 (23–43)	29 (27–46)	55 (39–64)	27 (23–41)	33 (26–50)
Elderly, age ≥60 y	37 (14)	27 (8)	4 (9)	3 (10)	8 (31)	4 (15)	83 (11)
Category of travelers							
Tourism	154 (58)	313 (95)	41 (95)	29 (97)	23 (88)	26 (100)	586 (81)
Business travelers	93 (35)	17 (5)	2 (5)	1 (3)	3 (12)	0 (0)	116 (16)
VFR	19 (7)	1 (<1)	0 (0)	0 (0)	0 (0)	0 (0)	20 (3)

Category	Africa n = 267 (%)	Asia n = 330 (%)	South America n = 43 (%)	Central America and the Caribbean n = 30 (%)	North America and Europe n = 26 (%)	Other§ n = 26 (%)	Total N = 722 (%)
Type of illness							
Potentially preventable	85 (32)	25 (8)	0 (0)	1 (3)	0 (0)	1 (4)	112 (16)
Febrile							
Unspecified febrile illness	34 (13)	55 (17)	7 (16)	3 (10)	6 (23)	4 (15)	109 (15)
Malaria							
P. falciparum‡	82 (30)	4 (1)	0 (0)	0 (0)	0 (0)	0 (0)	86 (12)
P. vivax	23 (9)	7 (2)	3 (7)	0 (0)	0 (0)	3 (12)	36 (5)
P. ovale	8 (3)	0 (0)	0 (0)	0 (0)	0 (0)	0 (0)	8 (1)
P. malariae	7 (3)	0 (0)	0 (0)	0 (0)	0 (0)	0 (0)	7 (<1)
Unidentified malaria	6 (2)	2 (<1)	0 (0)	0 (0)	0 (0)	0 (0)	8 (1)
Dengue fever	4 (1)	59 (18)	2 (5)	8 (27)	0 (0)	1 (4)	74 (10)
Enteric fever							
S. enterica ser. Typhi	2 (<1)	19 (6)	0 (0)	1 (3)	0 (0)	1 (4)	23 (3)
S. enterica ser. Paratyphi	0 (0)	36 (11)	0 (0)	0 (0)	0 (0)	0 (0)	36 (5)
Leptospirosis	1 (<1)	18 (5)	0 (0)	7 (23)	1 (4)	2 (8)	29 (4)
Pneumonia	10 (4)	11 (3)	0 (0)	1 (3)	5 (19)	1 (4)	28 (4)
Febrile diarrheal diseases							
Febrile diarrhea	7 (3)	4 (1)	0 (0)	0 (0)	0 (0)	1 (4)	12 (2)
Febrile gastroenteritis	2 (<1)	0 (0)	0 (0)	0 (0)	1 (4)	0 (0)	3 (<1)
Shigella sonnei	1 (<1)	1 (<1)	0 (0)	0 (0)	0 (0)	0 (0)	2 (<1)
Febrile dysentery	0 (0)	0 (0)	0 (0)	0 (0)	1 (4)	0 (0)	1 (<1)
Campylobacter jejuni	0 (0)	0 (0)	1 (2)	0 (0)	0 (0)	0 (0)	1 (<1)
Febrile non-diarrheal GI	1 (<1)	1 (<1)	0 (0)	0 (0)	0 (0)	1 (<1)	3 (<1)
Acute schistosomiasis	16 (6)	3 (<1)	0 (0)	0 (0)	0 (0)	0 (0)	19 (3)
Influenza	0 (0)	7 (2)	0 (0)	0 (0)	0 (0)	1 (4)	8 (1)
Epstein–Barr virus	1 (<1)	6 (2)	1 (2)	0 (0)	0 (0)	0 (0)	8 (1)
Cytomegalovirus	4 (1)	2 (<1)	2 (5)	0 (0)	0 (0)	0 (0)	8 (1)
Ameobic liver abscess	0 (0)	5 (2)	0 (0)	0 (0)	0 (0)	2 (8)	7 (<1)
Rickettsial diseases	3 (1)	3 (<1)	0 (0)	0 (0)	0 (0)	0 (0)	6 (<1)
Upper respiratory tract infection	4 (1)	1 (<1)	0 (0)	0 (0)	0 (0)	0 (0)	5 (<1)
Pyelonephritis	1 (<1)	3 (<1)	0 (0)	0 (0)	0 (0)	0 (0)	4 (<1)
Histoplasmosis	0 (0)	0 (0)	2 (5)	2 (7)	0 (0)	0 (0)	4 (<1)
Meningitis	2 (<1)	0 (0)	2 (5)	0 (0)	0 (0)	0 (0)	4 (<1)
Febrile eosinophilia	0 (0)	2 (<1)	1 (2)	0 (0)	0 (0)	0 (0)	3 (<1)
Endocarditis	1 (<1)	2 (<1)	0 (0)	0 (0)	0 (0)	0 (0)	3 (<1)
Legionella	1 (<1)	1 (<1)	0 (0)	0 (0)	1 (4)	0 (0)	3 (<1)
Febrile cellulitis	1 (<1)	1 (<1)	1 (2)	0 (0)	0 (0)	0 (0)	3 (<1)
Encephalitis	0 (0)	2 (<1)	0 (0)	0 (0)	1 (4)	0 (0)	3 (<1)
Brucella	1 (<1)	2 (<1)	0 (0)	0 (0)	0 (0)	0 (0)	3 (<1)
Coxiella burnetii	3 (1)	0 (0)	0 (0)	0 (0)	0 (0)	0 (0)	3 (<1)
Tuberculosis	2 (<1)	0 (0)	0 (0)	0 (0)	0 (0)	0 (0)	2 (<1)
HIV	0 (0)	0 (0)	0 (0)	0 (0)	1 (4)	0 (0)	1 (<1)
Other febrile							
Chikungunya	0 (0)	2 (<1)	0 (0)	0 (0)	0 (0)	0 (0)	2 (<1)
Melioidosis	0 (0)	2 (<1)	0 (0)	0 (0)	0 (0)	0 (0)	2 (<1)
Herpes Simplex virus 1 (HSV1)	0 (0)	2 (<1)	0 (0)	0 (0)	0 (0)	0 (0)	2 (<1)
Untyped infectious mononucleosis	0 (0)	2 (<1)	0 (0)	0 (0)	0 (0)	0 (0)	2 (<1)
Pericarditis	0 (0)	2 (<1)	0 (0)	0 (0)	0 (0)	0 (0)	2 (<1)
Ross River Fever	0 (0)	0 (0)	0 (0)	0 (0)	0 (0)	1 (4)	1 (<1)
Hantavirus	0 (0)	1 (<1)	0 (0)	0 (0)	0 (0)	0 (0)	1 (<1)
Trypanosomiasis	1 (<1)	0 (0)	0 (0)	0 (0)	0 (0)	0 (0)	1 (<1)
Febrile infected wound	0 (0)	0 (0)	1 (2)	0 (0)	0 (0)	0 (0)	1 (<1)
Respiratory Syncytial virus	0 (0)	1 (<1)	0 (0)	0 (0)	0 (0)	0 (0)	1 (<1)
Bronchitis	0 (0)	1 (<1)	0 (0)	0 (0)	0 (0)	0 (0)	1 (<1)
Meningoencephalitis	0 (0)	1 (<1)	0 (0)	0 (0)	0 (0)	0 (0)	1 (<1)
Meningoencephalitis d/t HSV2	0 (0)	1 (<1)	0 (0)	0 (0)	0 (0)	0 (0)	1 (<1)
Myocarditis	0 (0)	0 (0)	1 (2)	0 (0)	0 (0)	0 (0)	1 (<1)
Febrile inflammatory bowel disease	1 (<1)	0 (0)	0 (0)	0 (0)	0 (0)	0 (0)	1 (<1)
Afebrile conditions							
Afebrile diarrheal diseases							
Afebrile diarrhea	3 (1)	7 (2)	2 (5)	0 (0)	2 (8)	0 (0)	14 (2)
Afebrile gastroenteritis	1 (<1)	0 (0)	0 (0)	0 (0)	1 (4)	0 (0)	2 (<1)
Afebrile dysentery	1 (<1)	0 (0)	0 (0)	0 (0)	1 (4)	0 (0)	2 (<1)

Category	Africa n = 267 (%)	Asia n = 330 (%)	South America n = 43 (%)	Central America and the Caribbean n = 30 (%)	North America and Europe n = 26 (%)	Other§ n = 26 (%)	Total N = 722 (%)
Afebrile eosinophilia	4 (1)	6 (2)	2 (5)	1 (3)	1 (4)	2 (8)	16 (2)
Skin disease							
Afebrile cellulitis	3 (1)	2 (<1)	0 (0)	1 (3)	0 (0)	0 (0)	6 (<1)
Rash	2 (<1)	0 (0)	0 (0)	0 (0)	0 (0)	0 (0)	2 (<1)
Skin ulcer	1 (<1)	1 (<1)	0 (0)	0 (0)	0 (0)	0 (0)	2 (<1)
Afebrile infected wound	1 (<1)	0 (0)	0 (0)	0 (0)	0 (0)	0 (0)	1 (<1)
Pyoderma	0 (0)	1 (<1)	0 (0)	0 (0)	0 (0)	0 (0)	1 (<1)
Urticaria	0 (0)	0 (0)	1 (2)	0 (0)	0 (0)	0 (0)	1 (<1)
Miyasis	0 (0)	0 (0)	1 (2)	0 (0)	0 (0)	0 (0)	1 (<1)
Afebrile non-diarrheal GI							
Afebrile abdominal pain	2 (<1)	5 (2)	2 (5)	0 (0)	0 (0)	0 (0)	9 (1)
Other GI symptoms	0 (0)	3 (1)	0 (0)	0 (0)	0 (0)	0 (0)	3 (<1)
Vomiting alone	1 (<1)	0 (0)	0 (0)	0 (0)	0 (0)	0 (0)	1 (<1)
Viral hepatitis	2 (<1)	8 (2)	0 (0)	2 (7)	0 (0)	0 (0)	12 (2)
Leishmaniasis	2 (<1)	0 (0)	9 (21)	0 (0)	0 (0)	0 (0)	11 (2)
Giardiasis	0 (0)	4 (1)	0 (0)	1 (3)	0 (0)	0 (0)	5 (<1)
Neurocysticerosis	1 (<1)	3 (1)	0 (0)	0 (0)	0 (0)	0 (0)	4 (<1)
Filariasis	4 (1)	0 (0)	0 (0)	0 (0)	0 (0)	0 (0)	4 (<1)
Lymphadenopathy	1 (<1)	2 (<1)	0 (0)	0 (0)	0 (0)	1 (4)	4 (<1)
Deep vein thrombosis	0 (0)	1 (<1)	0 (0)	1 (3)	1 (4)	0 (0)	3 (<1)
Reactive arthritis	0 (0)	0 (0)	1 (2)	1 (3)	1 (4)	0 (0)	3 (<1)
Strongyloides	0 (0)	1 (<1)	0 (0)	0 (0)	0 (0)	2 (8)	3 (<1)
Other afebrile							
Onchocerciasis	2 (<1)	0 (0)	0 (0)	0 (0)	0 (0)	0 (0)	2 (<1)
Pulmonary embolism	0 (0)	2 (<1)	0 (0)	0 (0)	0 (0)	0 (0)	2 (<1)
Afebrile Inflammatory bowel disease	1 (<1)	0 (0)	0 (0)	0 (0)	1 (4)	0 (0)	2 (<1)
Cough	1 (<1)	1 (<1)	0 (0)	0 (0)	0 (0)	0 (0)	2 (<1)
Chest pain	1 (<1)	0 (0)	1 (2)	0 (0)	0 (0)	0 (0)	2 (<1)
Anemia	0 (0)	1 (<1)	0 (0)	0 (0)	0 (0)	0 (0)	1 (<1)
Henoch-Schonlein Purpura	0 (0)	1 (<1)	0 (0)	0 (0)	0 (0)	0 (0)	1 (<1)
Seizure	0 (0)	1 (<1)	0 (0)	0 (0)	0 (0)	0 (0)	1 (<1)
Helicobacter pylori	0 (0)	0 (0)	0 (0)	0 (0)	0 (0)	1 (4)	1 (<1)
Furunculosis	0 (0)	1 (<1)	0 (0)	0 (0)	0 (0)	0 (0)	1 (<1)
Afebrile URTI	0 (0)	1 (<1)	0 (0)	0 (0)	0 (0)	0 (0)	1 (<1)
Trauma	0 (0)	1 (<1)	0 (0)	0 (0)	0 (0)	0 (0)	1 (<1)
Muscle weakness	0 (0)	0 (0)	0 (0)	0 (0)	0 (0)	1 (4)	1 (<1)
Appendicitis	0 (0)	0 (0)	0 (0)	1 (3)	0 (0)	0 (0)	1 (<1)
Fatigue	0 (0)	1 (<1)	0 (0)	0 (0)	0 (0)	0 (0)	1 (<1)
Renal failure	0 (0)	1 (<1)	0 (0)	0 (0)	0 (0)	0 (0)	1 (<1)
Rheumatoid arthritis	0 (0)	1 (<1)	0 (0)	0 (0)	0 (0)	0 (0)	1 (<1)
Leg pain	1 (<1)	0 (0)	0 (0)	0 (0)	0 (0)	0 (0)	1 (<1)
Abdominal mass w eosinophilia	1 (<1)	0 (0)	0 (0)	0 (0)	0 (0)	0 (0)	1 (<1)
Idiopathic thrombocytopenic purpura	0 (0)	1 (<1)	0 (0)	0 (0)	0 (0)	0 (0)	1 (<1)
Tropical splenomegaly	1 (<1)	0 (0)	0 (0)	0 (0)	0 (0)	0 (0)	1 (<1)
Coccidiosis	0 (0)	0 (0)	0 (0)	0 (0)	1 (4)	0 (0)	1 (<1)
Echinococcosis	0 (0)	0 (0)	0 (0)	0 (0)	0 (0)	1 (4)	1 (<1)
Unidentified helminth	0 (0)	1 (<1)	0 (0)	0 (0)	0 (0)	0 (0)	1 (<1)
Outcome							
Intensive care unit hospitalization	4 (1)	4 (1)	0 (0)	0 (0)	2 (8)	1 (4)	11 (2%)
Mortality	1 (<1)	1 (<1)	0 (0)	0 (0)	0 (0)	0 (0)	2 (<1%)

*IQR, interquartile range; VFR, visiting friends or relatives; URTI, upper respiratory tract infection; GI, gastrointestinal.
†Values are no. (%) except as indicated.
‡One patient returning from Asia with malaria had a coinfection with both *P. falciparum* and *P. vivax*. In this case, we decided to list the patient under *P. falciparum*.
§"Other" comprises both Oceania (n = 6) and cases whose exact region of infection was undetermined (n = 26).

Technical Appendix Table 2. Number of hospitalized Israeli travelers at Sheba Medical Center by destination country and United Nations World Tourism Organization estimates of Israeli traveler entries to the destination country, 2004–2015 (n = 535)*†.

Continent	Country§	Number of hospitalizations	Israeli entries to country	Most common cause of hospitalization
Africa	Ethiopia‡	48	92,839	*Plasmodium vivax* (n = 21, 44%)
	Angola‡	38	28,862	*Plasmodium falciparum* (n = 16, 42%)
	Equatorial Guinea	33	NA	*Plasmodium falciparum* (n = 19, 58%)
	Nigeria	24	225,824	*Plasmodium falciparum* (n = 9, 38%)
	Tanzania, United Republic of	16	52,651	Schistosomiasis (n = 4, 25%),
	Uganda	15	14,786	*Plasmodium falciparum* (n = 6, 40%)
	Congo, Democratic Republic of the	12	24,464	*Plasmodium falciparum* (n = 4, 33%)
	Sierra Leone	8	1,644	*Plasmodium falciparum* (n = 4, 50%)
	Ghana	8	NA	Diarrhea (n = 3, 38%)
	Liberia	7	NA	*Plasmodium falciparum* (n = 5, 71%)
Asia	India	116	542,915	Dengue fever (n = 17, 15%)
	Thailand	106	1,410,957	Dengue fever (n = 36, 34%)
	Nepal	37	72,025	*S.enterica* ser. Paratyphi (n = 23, 62%)
	China	12	1,329,533	Unspecified febrile disease (n = 2, 17%)
	Lao People's Democratic Republic	8	45,465	Leptospirosis (n = 4, 50%)
Americas	Bolivia	9	NA	Leishmania braziliensis (n = 9, 100%)
	Guatemala	8	NA	Leptospirosis (n = 4, 50%)
	Costa Rica	7	125,237	Leptospirosis (n = 4, 57%)
	Peru	7	144,427	Afebrile non-diarrheal GI (n = 2, 29%) Cytomegalovirus (n = 2, 29%)
	United States	6	3,799,836	Single cases of deep vein thrombosis, encephalitis, Coccidiosis, gastroenteritis, pneumonia, leptospirosis.
	Brazil	5	410,326	Unspecified febrile disease (n = 2, 40%)
**Oceania**	Papua New Guinea	5	2,047	Leptospirosis (n = 2, 40%) *Plasmodium vivax* (n = 2, 40%)

*UNWTO, United Nations World Tourism Organization (1); NA, not applicable.
†Included are all countries with ≥5 hospitalizations regardless of whether they consistently reported to UNWTO; when reporting was inconsistent, we denote NA for number of entries.
‡For Angola and Ethiopia, one year of Israeli citizen entry data was missing. Assuming the number of entries did not change substantially during the missing year, we used the average number of entries during all other years as the number of entries during in the missing year.
§Countries that officially forbid entry of Israeli citizens: Algeria, Bangladesh, Brunei, Iran, Iraq, Kuwait, Lebanon, Libya, Malaysia, Oman, Pakistan, Saudi Arabia, Sudan, Syria, United Arab Emirates, Yemen.

Reference

1. United Nations World Tourism Organization. Israel: Country-specific: Outbound tourism 1995–2016 (12.2017). Tourism statistics. 2017. http://www.e-unwto.org/doi/abs/10.5555/unwtotfb0376250119952016201712
